# CircRNA0001859, a new diagnostic and prognostic biomarkers for COPD and AECOPD

**DOI:** 10.1186/s12890-020-01333-1

**Published:** 2020-11-25

**Authors:** Shuifang Chen, Yinan Yao, Shan Lu, Junjun Chen, Guangdie Yang, Lingfang Tu, Lina Chen

**Affiliations:** The First Affiliated Hospital, College of Medicine, Zhejiang University, NO.79, Qingchun Road, Hangzhou, 310003 China

**Keywords:** Circular RNA0001859 (CircRNA0001859), CS, NNK, Chronic obstructive pulmonary disease (COPD), Acute exacerbation of COPD (AECOPD), Lung cancer

## Abstract

**Background:**

Dysregulation of circRNAs has been reported to be functionally associated with chronic obstructive pulmonary disease (COPD). The present investigation elucidated the potential role of CircRNA0001859 in regulating chronic obstructive pulmonary disease acute (COPD) and Acute Exacerbation of COPD (AECOPD).

**Methods:**

Mice model of COPD was established to screen and verify the dysregulated expression of CircRNA0001859. Fluorescence in situ hybridization (FISH) and quantitative real-time PCR (qRT-PCR) were carried out to detect the expression of CircRNA0001859. 38 stable COPD patients, 24 AECOPD patients, 57 COPD with lung cancer patients and 28 healthy person with age and sex matched to total patients were used for the present investigation.

**Results:**

circRNA0001859 was downregulated in the lung tissue of mice after the three kinds of treatments (Cigarette smoke (CS)/NK alone or CS + NNK) for inducing COPD. FISH assay verified the downregulation of circRNA0001859 both in the mice lung and human bronchial epithelial cell of COPD model. Furthermore,, the level of circRNA0001859 was also downregulated in the peripheral blood of COPD and lung cancer patients. CircRNA0001859 might act as a diagnostic and prognostic biomarker for the treatment of in COPD and AECOPD with Are under the receiver operating characteristic curve (ROC curve) (AUC) of 0.7433 and 0.8717, respectively.

**Conclusion:**

We explored a novel circRNA0001859, which might act as a potential therapeutic biomarker for the treatment of COPD and AECOPD.

## Background

Chronic obstructive pulmonary disease (COPD), a heterogeneous disorder is estimated to be the third leading cause of chronic morbidity and mortality worldwide [1]. COPD is associated with severe coronavirus disease 2019 (COVID-19) [2]. Global Burden of Diseases, Injuries and Risk factors study (GBD) 2017, reported that COPD lead to an estimated death of 3.2 million people worldwide [3]. China Pulmonary Health (CPH) study on 50,991 individuals (21,446 men and 29, 545 women) with reliable post-bronchodilator results revealed prevalence of spirometry-defined COPD to be 8.6% which is accounting for 99.9 million people with COPD in China and seen higher COPD rates in men (11.9%) than in women (5.4%) and in people aged 40 years or older (13.7%) than in those aged between 20 and 39 years; the major risk of COPD in Chinese population includes cigarette smoking, ambient air pollution, underweight, childhood chronic cough, parental history of respiratory disease and lower education [4].

Circular RNAs (CircRNAs) are evolutionarily conserved noncoding RNAs; have unique closed loop structure formed by reverse splicing and subsequent removal of 5′ cap and 3′ poly-A tail. They have higher stability than the linear RNAs as they are not digested by RNase R exonuclease [5]. They are abundant and have high organ specificity [6]. CircRNAs for the first time are observed in the early 1970s in plant viroid and they were considered as transcriptional “noise” with no biological function but the recent emerging evidences have shown that it plays a crucial role in physiological and pathological processes and due to their abundance and stability [7–9]. They have been considered as potential prognostic and diagnostic biomarkers in diverse range of diseases [10]. CircRNAs regulate these biological processes by sponging miRNAs, protein coding, alternative splicing, transcriptional and posttranscriptional regulations inhibit cell cycle protein bait function and circRNA derived pseudogenes [11, 12].

CircRNAs have been proved to be involved in respiratory diseases, such as lung cancer acute respiratory distress syndrome, pulmonary hypertension pulmonary tuberculosis and silicosis [13, 14]. Also, circRNA are associated with COPD. The has_circ_0016070 is associated with vascular remodeling in pulmonary arterial hypertension (PAH) in COPD patients by promoting the proliferation of pulmonary artery smooth muscle cells (PASMCs) via the miR-942/Cyclin D1 (CCND1) axis; has_circ_0016070 significantly increased cell viability and decreased the number of cells arrested in the G1/G0 phase [15]. The has_circ_000360 is significantly downregulated in the primary human small-airway epithelial cells (HSAECs) model of cigarette smoke extract induced COPD [16]. However, the underlying mechanism of circRNA in COPD and AECOPD remains largely unknown.

In the present investigation, we have explored the potential role of a novel circRNA, circ0001859 in COPD and AECOPD. Our results have demonstrated that circ0001859 might act as a potential biomarker for diagnostic and therapeutic target to treat COPD and AECOPD.

## Methods

### Ethics statement

This study was approved by the Ethics Committee of the First Affiliated Hospital, College of Medicine, Zhejiang University, China. We have taken written informed consent from participants prior to their enrollment in the study.

Animals, Diet and Study Groups 28 healthy persons, 38 stable COPD patients, 24 AECOPD patients,57 COPD complicating lung cancer patients were recruited from the First Affiliated Hospital, College of Medicine, Zhejiang University between December 1, 2014 to March 31, 2016. These groups were further classified into the following [17]:

1. Healthy controls (HCs): HCs were selected with age, gender, and body mass index (BMI) matched to total COPD patients. HCs with any other co-existing diseases, such as infection, lung diseases, renal or hepatic dysfunction and history of severe infection, solid tumor, hematological diseases, and autoimmune diseases were excluded from this study.

2. AECOPD patients: The inclusion criteria of AECOPD patients were: age > 40 years; diagnosed with COPD according to Global Initiative for Chronic Obstructive Lung Disease (GOLD) criteria, USA; increased dyspnea and decreased performance in daily activities, altered amount and color of sputum, increased coughing, pyrexia and/or altered mental status.

The exclusion criteria were: Patients with complicated asthma, lung cancer, or other relevant lung diseases; history of severe infection, malignant tumors, and autoimmune diseases were excluded.

3. Stable COPD patients: The inclusion criteria of stable COPD patients were as follows: age > 40 years; diagnosed with COPD according to GOLD criteria; without acute exacerbation in the last 6 months. The exclusion criteria were similar to AECOPD patients.

4. Lung cancer patients complicated with COPD: lung cancer patients complicated with COPD symptoms were those pathologically confirmed and diagnosed as lung cancer, meantime, diagnosed as COPD before.

### Cigarette smoke extract (CSE) preparation

Cigarette was combusted through a 50 mL syringe apparatus. CSE was obtained by combustion of two filter less cigarettes per trial and subsequently bubbled via bubbling the smoke of through 10 mL of Dulbecco’s Modified Eagle Medium (DMEM) for 2 min. The solution was neutralized with 1 M NaOH (pH 7.4) and then sterilized through a glass ball filter of pore size, 0.22 μm. The smoked medium was considered 100% CSE and diluted with Roswell Park Memorial Institute (RPMI 1640) medium (HyClone, USA) to obtain the desired concentration [18].

### Cell culture

Human bronchial epithelial cell line (BEAS-2B) was purchased by American Type Culture Collection (ATCC), USA. BEAS-2B cells were cultured in RPMI 1640 medium (HyClone, USA) containing 10% fetal bovine serum (FBS, HyClone, USA) supplemented with100 mg/L streptomycin, and 100 U/mL penicillin. The cells were incubated at 37 °C under 5% CO_2_ and saturated humidity.

### COPD model establishment

A total of 40 adult male C57BL/6 mice (age:6–8 weeks; weight: 18–20 g) were obtained from the Animal Center, of Zhejiang University and randomly divided into 4 groups. Each group has 10 mice. They were housed at 24 ± 1 °C with 50% relative humidity under 12 h light/dark cycle and were allowed to standard mouse food and water in environmentally controlled pathogen-free conditions during all the experiments. All protocols were approved by the Animal Experimental Ethics Committee, of Zhejiang University. The mice in cigarette smoke (CS) group were exposed to the smoke of four cigarettes without filter via a TE-10 smoking machine as previously described (Jiao Zi, Chengdu, China). The mice were placed in a 60 × 57 × 100 cm fume box and exposed to CS four time per day with 30 min intervals. Mice were exposed to the smoke 5 days/week for 14 weeks [19]. The mice in NNK group was injected of 4-(Methylnitrosamino)-1-(3-pyridyl)-1-butanone (NNK) (Toronto Research Chemicals, Canada) at a dose of 30 mg/kg body weight (BW) once a month for four consecutive months (a total dose of 120 mg/kg BW). In the CS + NNK treatment group, mice were grown under both CS and NNK. At the end of the study, the mice were subjected to euthanasia by injection of pentobarbital sodium (100 mg/kg).

### Enzyme linked immunosorbent assay (ELISA) assay

The levels of cytokines, including malondialdehyde (MDA), glutathione (GSH), Superoxide dismutase (SOD), Interleukin 10 (IL-10), Interleukin 6 (IL-6), Tumor necrosis factor alpha (TNF-a) in the bronchoalveolar lavage fluid, or cell supernatant were measured with the ELISA kits (MDA, S0131S, Beyotime, China; GSH, S0053, Beyotime, China; SOD, S0101M, Beyotime, China; IL-10, PI523, Beyotime, China; IL-6, sb-2506, Senbeijia, China; TNF-a, sb-2386, Senbeijia, China) according to the manufacturer’s instructions. Three independent biological repeats were used for each analysis. All samples were stored at − 80 °C before conducting the experiment.

### Bronchoalveolar lavage fluid (BALF) collection and cell count

Bronchoalveolar lavage fluid experiment was performed on the left lung after ligating the right lung. Repeated lavage was performed with 0.4 mL phosphate-buffered saline and fluid was collected thrice. The recovery rate was above 80%. The lavage fluid was collected in 1.5 mL tubes (placed on ice). Absolute cell counts were determined using a MEK-7222 k automatic hematology analyzer (Nihon Kohden, Tokyo, Japan). For the ELISA assay, the BALF solution was mixed well and centrifuged (4000×g, 10 min, 4 °C). The supernatant was aspirated into 1.5 mL tubes for the further research.

### Lung function investigation

PLY3211 lung function analysis system (BuxcoResearch Systems, USA) was applied for lung function measurement of all the mice used in the present study before sacrifice. Mice were anesthetized with pentobarbital (40 mg/kg), tracheostomized, and then put into the body chamber of the system. The average breathing frequency was set at 150 breaths/min. Parameters of pulmonary function such as FEV0.1, Cydn and Raw were measured.

### Hematoxylin-eosin (HE) staining

The lung tissues were resected and fixed in 10% formalin for 24 h. Then, these tissues were transferred to 75% ethanol, embedded and sectioned into 5 μm thickness. The sections on glass slides were dried at 60 °C for 15 min, and then deparaffinized and stained with hematoxylin and eosin (HE) by incubating the tissue sections in Harris hematoxylin (C0105S, Beyotime, Shanghai, China) followed by serial eosin (Beyotime, Shanghai, China). The sections were subjected to graded ethanol steps and finally, neutral gum sealing piece was performed.

### RNA extraction

Peripheral blood samples of healthy controls and patients were collected and stored at room temperature for few minutes. Subsequently, samples were centrifuged at 3000 rpm, 4 °C for 15 min and stored at − 80 °C for further experiments. RNA from serum, HBE cells and mouse lung tissues was extracted using a TRIzol Plus RNA purification kit (N065, Jiancheng biotech, Nanjing, China). The quality of RNA samples and small RNA fraction was checked via the Nanodrop spectrophotometer (Thermo, DE, USA). The primer were as follows: circRNA0001859 forward: GCCATTGTAGAAGCTGGTGG, reverse: AGCTGCACTAGAGTCCCAAG; U6 forward:CGCTTCACGA ATTTGCGTGTCAT, reverse: GCTTCGGCAGCACATATACTAAAAT.

### Immunohistochemistry (IHC)

The sections were inactivated with 3% hydrogen peroxide for 10 min at room temperature. The sections were blocked with 5% bovine serum albumin (BSA) for 20 min and incubated with anti-ki67 polyclonal antibody (1: 2000, abcam, England) at 4 °C overnight. Then, the sections were incubated with the Horseradish Peroxide (HRP)-conjugated secondary antibody at room temperature for 2 h. Then, the targets were visualized by diaminobenzidine (DAB, P0202, Beyotime, Shanghai, China) and counterstained with hematoxylin. Images of stained lung tissue sections were captured with an inverted fluorescence microscope (Olympus, Japan) and analyzed with Image Pro Plus 6.0 software (Media Cybernetics, Inc., Bethesda, MD, USA).

### MicroRNA array

After RNA extraction, the concentration of RNA was determined using NanoDrop system. Afterwards, the samples were then labeled using the miRCURY™ Hy3™/Hy5™ Power Labeling Kit (Exiqon, MA, USA) and hybridized with the miRCURY™ LNA MicroRNA Arrays (v.16.0, Exiqon). Following the washing steps, the slides were scanned using the Axon GenePix 4000B microarray scanner (Axon Instruments, CA, USA).

### Reverse transcription and quantitative real-time PCR

RNA extraction were performed with TRIzol reagent (15596–026, Invitrogen, USA), precipitated with isopropanol, washed with 75% ethanol and dissolved in RNase free water. Reverse transcription were performed with 1 μg RNA using the cDNA transcription kit (AE301–02, TransGen Biotech Co. Ltd., Beijing, China). 20 ng cDNA was used for qRT-PCR to validate the expression of relative mRNA expressions; it was determined by using SYBR green mix (Y-2744, Yisheng, Shanghai, China). The relative gene expression was evaluated based on the 2–ΔΔCt method.

### Fluorescence in situ hybridization (FISH) assay

Alexa Fluor 555-labeled circRNA0001859 probes were designed and synthesized by RiboBio (Guangzhou, China). FISH experiment was carried out with a FISH Kit (lnc1100621, RiboBio, Guangzhou, China). 1 × 10^5^ cells were seed onto autoclaved glass slides and cultured for 24 h. After fixing with 4% paraformaldehyde for 20 min followed by permeabilization with 0.5% Triton X-100 for 10 min, the cells were cultured at 37 °C overnight. Finally, the slides were incubated with DAPI to stain cell nucleus and observed them under a fluorescence microscope (Leica, Wetzlar, Germany).

### Statistical analysis

SPSS 20.0 software (SPSS Inc., Chicago, IL, USA) were used to analyze all data for statistical significance. All the data are presented as the means ± SD. Unpaired and paired t-tests were used for the statistical analysis. A receiver operating characteristi ccurve (ROC) was drawn to assess diagnostic accuracy. Spearman’s correlation analysis was used to evaluate the correlation between circRNA0001859 and risk factors. *P* < 0.05 was considered as statistical significance. Three independent biological repeats were used for each analysis.

## Results

A total of 147 individuals, including 28 Healthy controls (HC), 38 COPD, 24 AECOPD and 57 COPD patients with lung cancer were took part in the present study (Table 1). There was no difference in age, gender, and body mass index (BMI) for the abovementioned four groups. However, the values of Forced Expiratory Volume in 1 second (FEV1) (L), FEV1/Forced Vital Capacity (FVC) (%) and FEV1% predicted in COPD, AECOPD and COPD patients with lung cancers were remarkably reduced as compared to the HC group (*P* < .001) (Table 1).

**Table 1:** Baseline characteristics of health control, stable COPD patients, AECOPD patients and lung cancer patients

Parameter	Health Control(28)	COPD Patients(38)	AECOPD Patients(24)	COPD complicating Lung cancer (57)
Age (year)	67.1 ± 10.2	67.4 ± 6.3	68.6 ± 8.1	69.6 ± 9.4
Gender (male/female)	18/10	22/16	15/9	36/21
BMI (kg/m2)	22.8 ± 4.1	22.1 ± 3.3	22.6 ± 2.8	22.1 ± 3.8
Smoke history (Yes/No)	11/17	22/16	15/9	31/26
Type of cancer	/	/	/	Non-small cell lung cancer
FEV1 (L)	1.99 ± 0.41	0.97 ± 0.45^*^	1.00 ± 0.47^*^	0.98 ± 0.37^*^
FEV1(% predicted)	93.6 ± 7.4	42.2 ± 14.9^*^	44.2 ± 13.8^*^	41.5 ± 5.8^*^
FEV1/FVC (%)	77.6 ± 3.1	47.8 ± 13.2^*^	49.5 ± 11.3^*^	47.8 ± 8.2^*^

*p<0.05

### CircRNA0001859 was down-regulated in lung tissue of COPD mouse

**Fig. 1:**
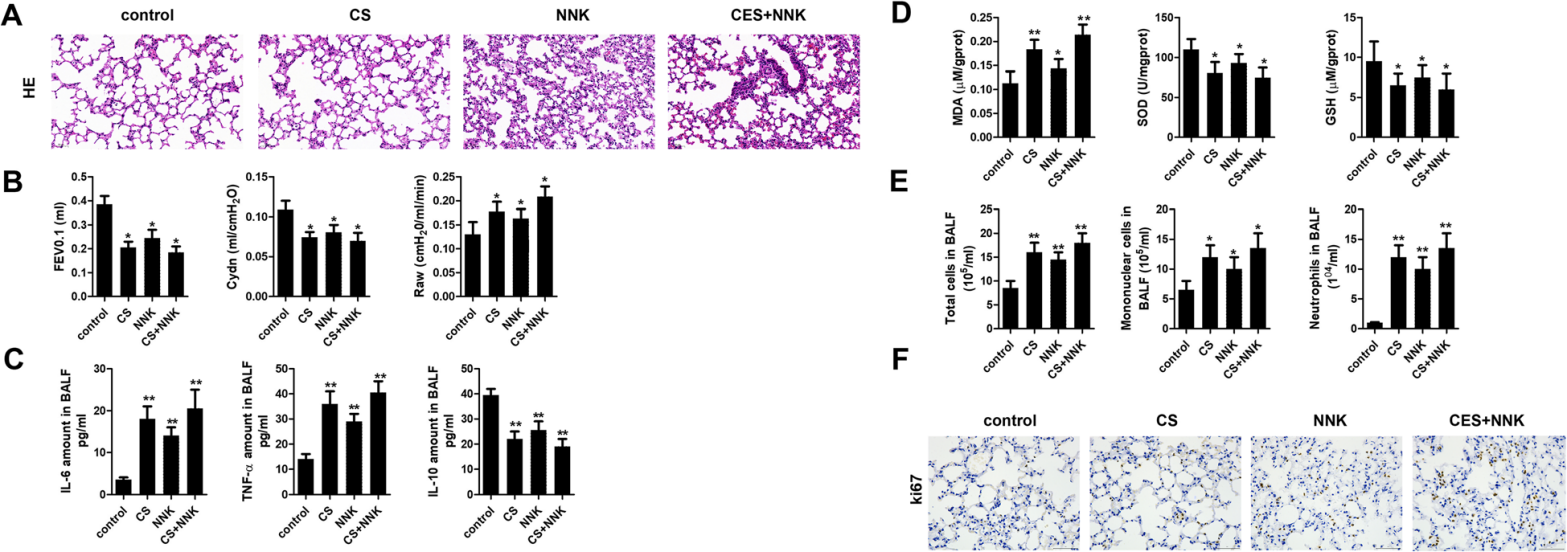
The evaluation of the animal model establishment. **a** HE staining was used to detect the pathological changes in lung tissues (× 200, *n* = 8). **b** Lung function parameters including FEV0.1, Cydn and Raw were evaluated. **c** Inflammatory cytokines such as IL-6, TNF-α and IL-10 in BALF were detected using ELISA (*n* = 8). **d** Oxidative stress related cytokines including MDA, SOD and GSH were evaluated using ELISA assay (*n* = 8). **e** Cell counts of total cell, mononuclear cell and neutrophils in BALF were carried out (*n* = 8). **f**. IHC was performed to evaluate the expression of ki67 in lung tissue (× 100, *n* = 8). *p < 0.05, **p < 0.01. Three independent biological repeats were carried out for each analysis

**Fig. 2:**
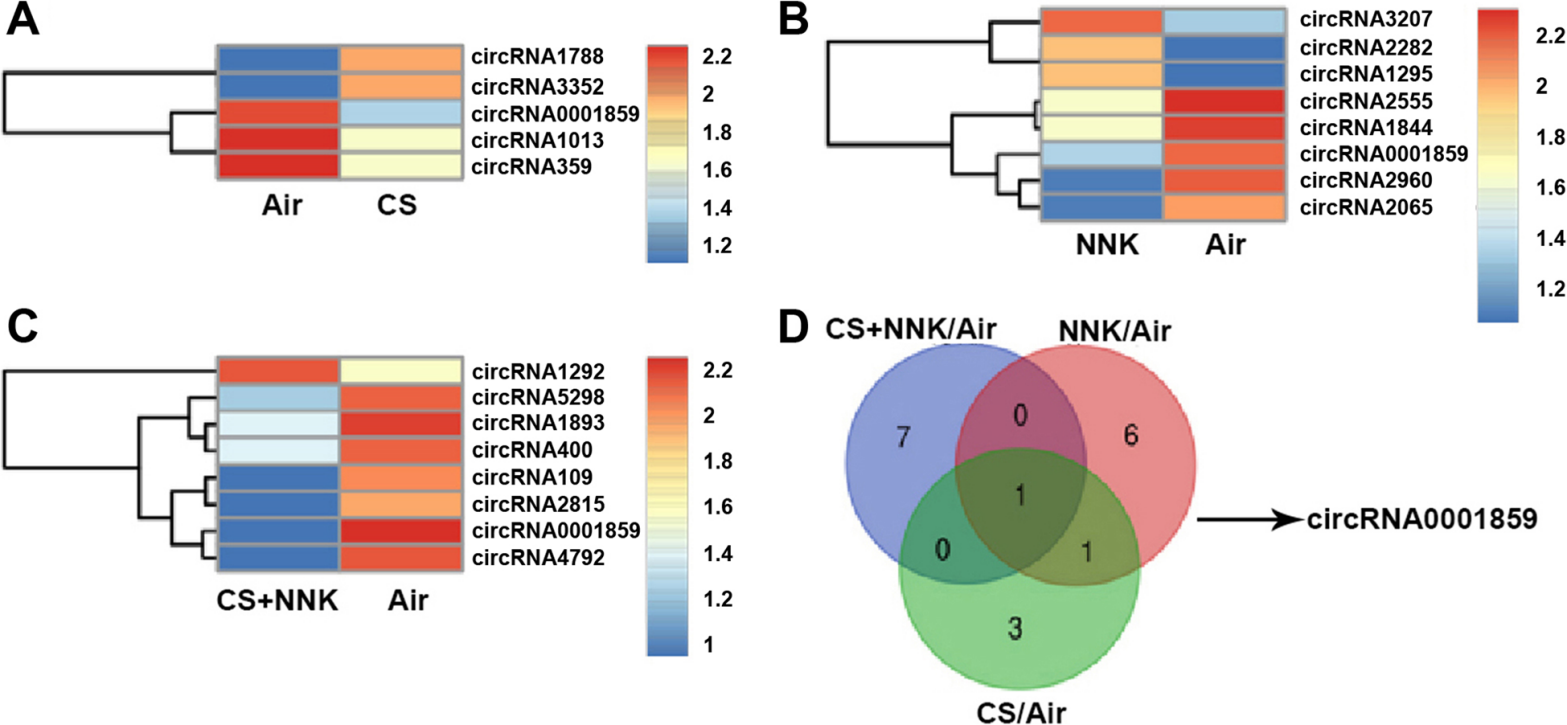
CircRNA0001859 was down-regulated in lung tissue of COPD mouse. **a** The dysregulated expression of circRNAs in CS treatment group as compared with the Air treatment group. **b** The dysregulated expression of circRNAs in NNK treatment group as compared with the Air treatment group. **c** The dysregulated expression of circRNAs in CS + NNK treatment group as compared with the Air treatment group. **d** The Venn diagram showed the dysregulated circRNA after all the three kinds of treatments (CS/NK alone or CS + NNK). Three independent biological repeats were carried out for each analysis

**Fig. 3:**
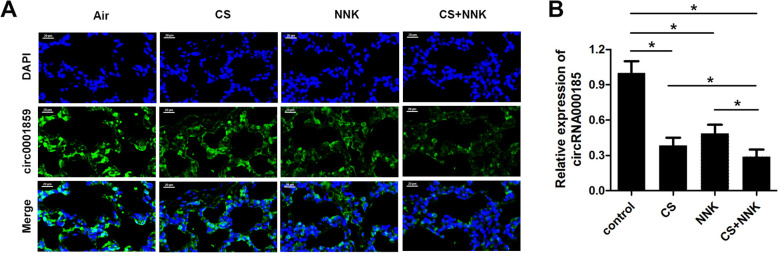
CircRNA0001859 was downregulated in lung tissue of COPD mouse. **a** FISH assay with Alexa Fluor 555-labeled circRNA0001859 probe was performed to evaluate the expression of circRNA0001859 in the lung tissue (× 200, *n* = 6). **b** qRT-PCR was used to evaluate the expression of circRNA0001859 in the lung tissue (*n* = 6). *p < 0.05. Three independent biological repeats were carried out for each analysis

To investigate the role of circRNA in the progression of COPD, we established the mouse model of COPD using cigarette smoke (CS) treatment lasting for 14 weeks. Notably, tobacco carcinogen, NNK was used to establish the transformation from COPD to lung cancer (TFCLC) model for investigating the potential role of circRNA0001859 in TFCLC. Firstly, we verified the sensitivity of our model system using several experiments. HE staining showed that CS/NNK alone treatments for 1 month significantly increase the alveolar volume of mice. Increase in the rupture of large, alveolar septum and infiltration of inflammatory cell, indicated that CS/NNK alone might induce early COPD models. CS + NNK treatment of mice for 1 month dramatically increased the alveolar volume, severe alveolar fracture and invasion of more inflammatory cells (Fig. 1a). Afterwards, we evaluated the lung function; CS/NNK alone or CS + NNK treatments all of them significantly reduced the forced expiratory volume in the first 0.1 s (FEV0.1) and dynamic lung compliance (Cydn) while elevated high airway resistance (Raw) (Fig. 1b). In addition, CS/NNK alone or CS + NNK triggered the release of cytokine IL-6, TNF-α and malondialdehyde (MDA) while decreased in the level of IL-10, superoxide dismutase (SOD) and glutathione (GSH) (Fig. 1c, d). Furthermore, we evaluated the cell count by BALF analysis. We found that CS/NNK alone or CS + NNK increased the total number of cells, mononuclear cells and neutrophils (Fig. 1e), indicating that CS or NNK alone or CS + NNK treatments apparently lead to COPD phenotype and successful establishment of the COPD model. ki67, a prognostic and predictive indicator of tumor cell proliferation, staining of lung tissue indicated the transformation from COPD to lung cancer (Fig. 1f). Afterwards, we screened the expression of circRNAs in these three treatment groups of lung tissues by microarray analysis. Heat map two dimensional analysis indicated the dysregulated circRNAs (Fig. 2a-c). Finally, we selected circRNA0001859 by intersection of the three sets (Fig. 2d). To verify our microarray results, we performed FISH assay and qRt-PCR to detect the expression of circRNA0001859 using a specific probe. CircRNA0001859 was significantly down-regulated in the lung tissue of mice in CS/NNK alone or CS + NNK treatment groups (Fig. 3a and b). Moreover, the level of circRNA0001859 is lower in CS + NNK as compared to the CS/NNK alone. These results validated that circRNA0001859 possibly involved in the progression of COPD and to a limited extent in regulating the lung cancer.

### Downregulation of CircRNA0001859 in human bronchial epithelial cell subjected to CS/NNK alone or CS + NNK treatments

**Fig. 4:**
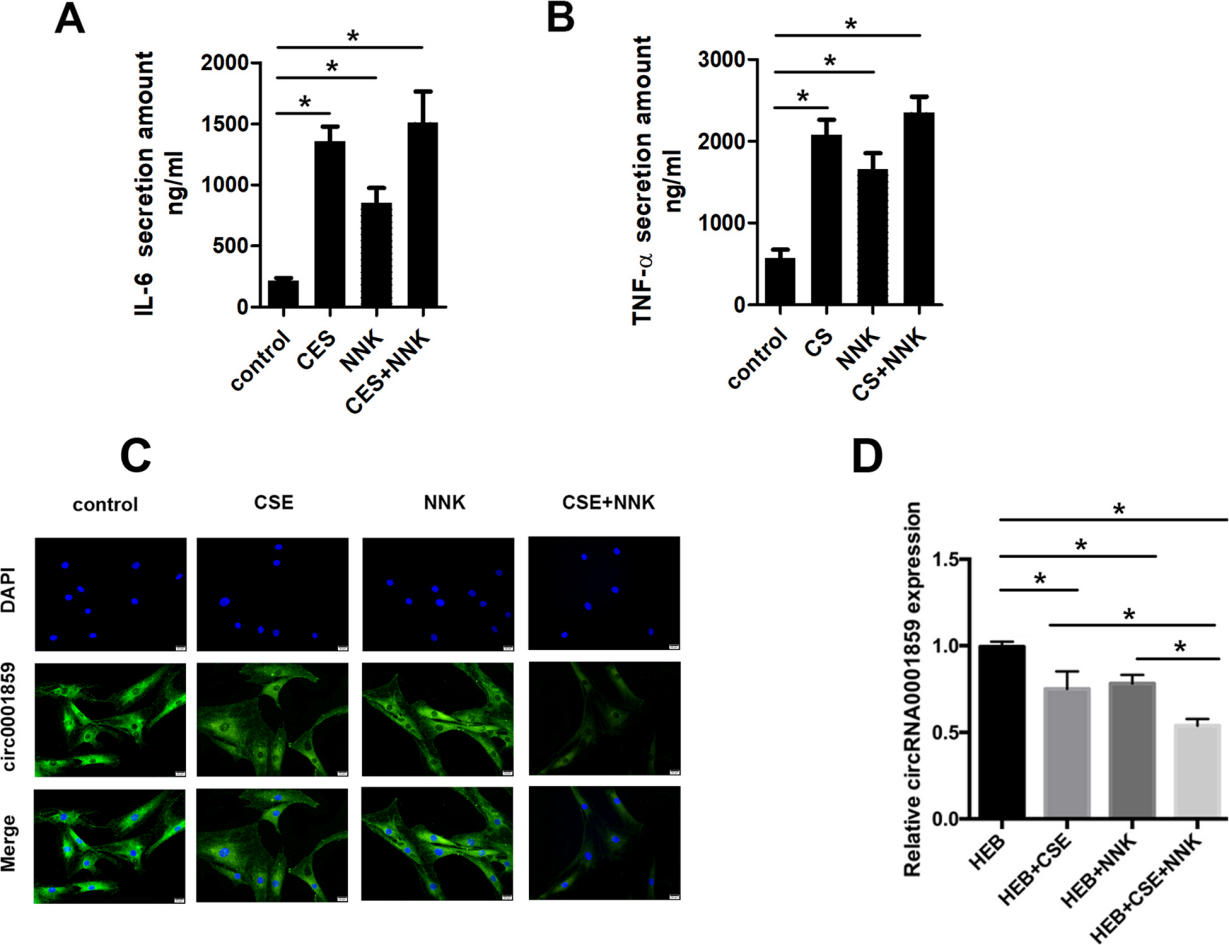
CircRNA0001859 was downregulated in human bronchial epithelial cell subjected to CS/NNK or CS + NNK treatment. FISH assay was performed to evaluate the expression of circRNA0001859 in the human bronchial epithelial cell subjected to three different treatments (× 200, *n* = 6). **b** qRT-PCR was used to evaluate the expression of circRNA0001859 in the lung tissue (*n* = 6). *p < 0.05. Three independent biological repeats were carried out for each analysis

As circRNA0001859 was significantly down-regulated in the lung of mice COPD therefore, we speculated whether it was dysregulated in human COPD or not. We treated human bronchial epithelial with CS/NNK or CSE + NNK to mimic human COPD in vitro; higher release of inflammatory cytokines, such as IL6 and TNF-α (Fig. 4a, b). qRT-PCR and FISH analyses showed down regulation of circRNA0001859 in CS/NNK alone as compared to the control group (Fig. 4c and d) Similar to mice-COPD, the expression of circRNA0001859 was lower in CSE + NNK combined group as compared with the CS/NNK treatment alone.

CircRNA0001859 was downregulated in serum obtained from in COPD, AECOPD and COPD patients with lung cancer and mice serum CS or NNK alone and CS + NNK treatments.

**Fig. 5:**
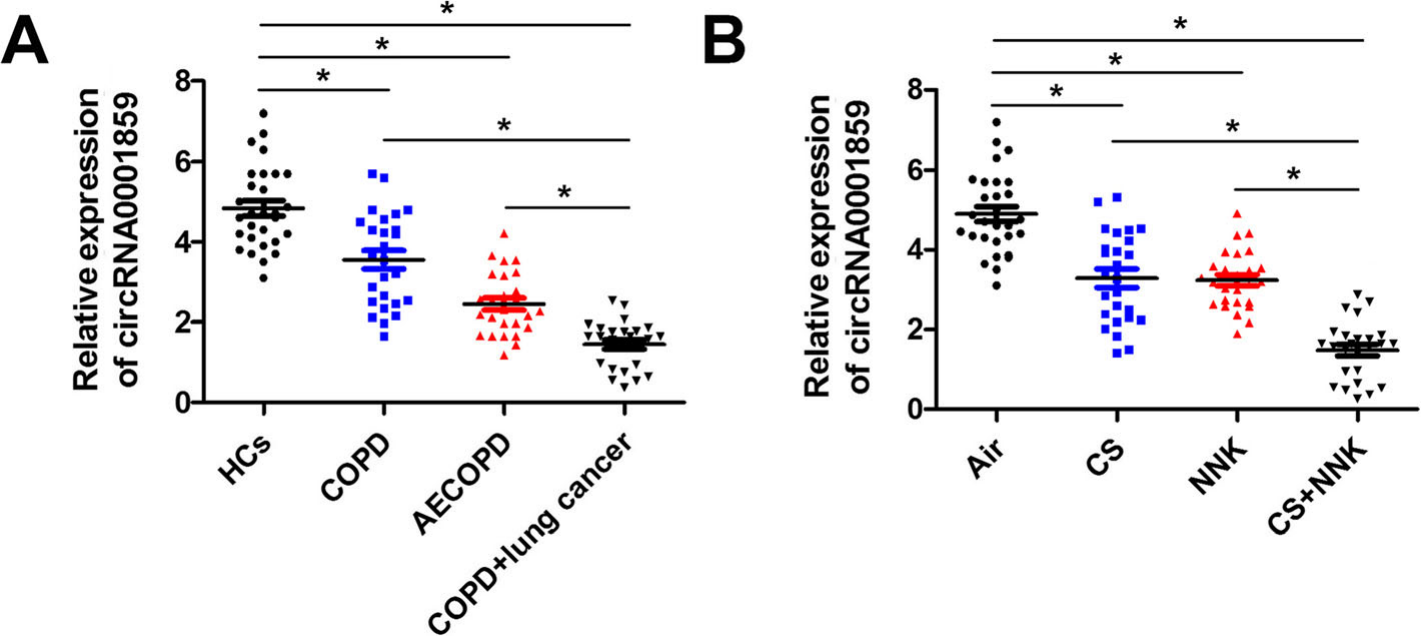
CircRNA0001859 was downregulated in serum obtained from COPD, AECOPD and lung cancer with COPD patients and mice serum treated by CS/NNK alone or CS + NNK. **a** IL-6 and **b** TNF-α were evaluated using ELISA (*n* = 6). qRT-PCR was performed to evaluate the expression of circRNA0001859 in the blood of patients (**c**) or mice (**d**) (*n* = 6). *p < 0.05. Three independent biological repeats were carried out for each analysis

CircRNAs are more stable than linear RNAs and therefore can be used as stable prognostic and diagnostic markers. The dysregulated expression of circRNA0001859 in mouse and bronchial epithelial cell model of COPD suggested us whether this circRNA expression was altered in serum. Next, we evaluated the level of circRNA0001859 of serum obtained from HC, COPD, AECOPD and COPD complicating lung cancer patients. Interestingly, circRNA0001859 was downregulated in the serum from in COPD, AECOPD and COPD patients with lung cancer as compared to the healthy controls (Fig. 4a). Furthermore, the level of circRNA0001859 in COPD complicating lung cancer patients was lower than that in normal COPD or AECOPD ipatients (Fig. 5a). Additionally, we also evaluated the circRNA0001859 level in serum of mouse. Consistently, expression level of circRNA0001859 was reduced in the CS/NNK alone as compared with the control group. Moreover, the level of circRNA0001859 in CS + NNK was significantly lower than that in the CS/NNK alone (Fig. 5b).

Proportions of serum circRNA0001859 in HCs, stable COPD and AECOPD.

**Fig. 6:**
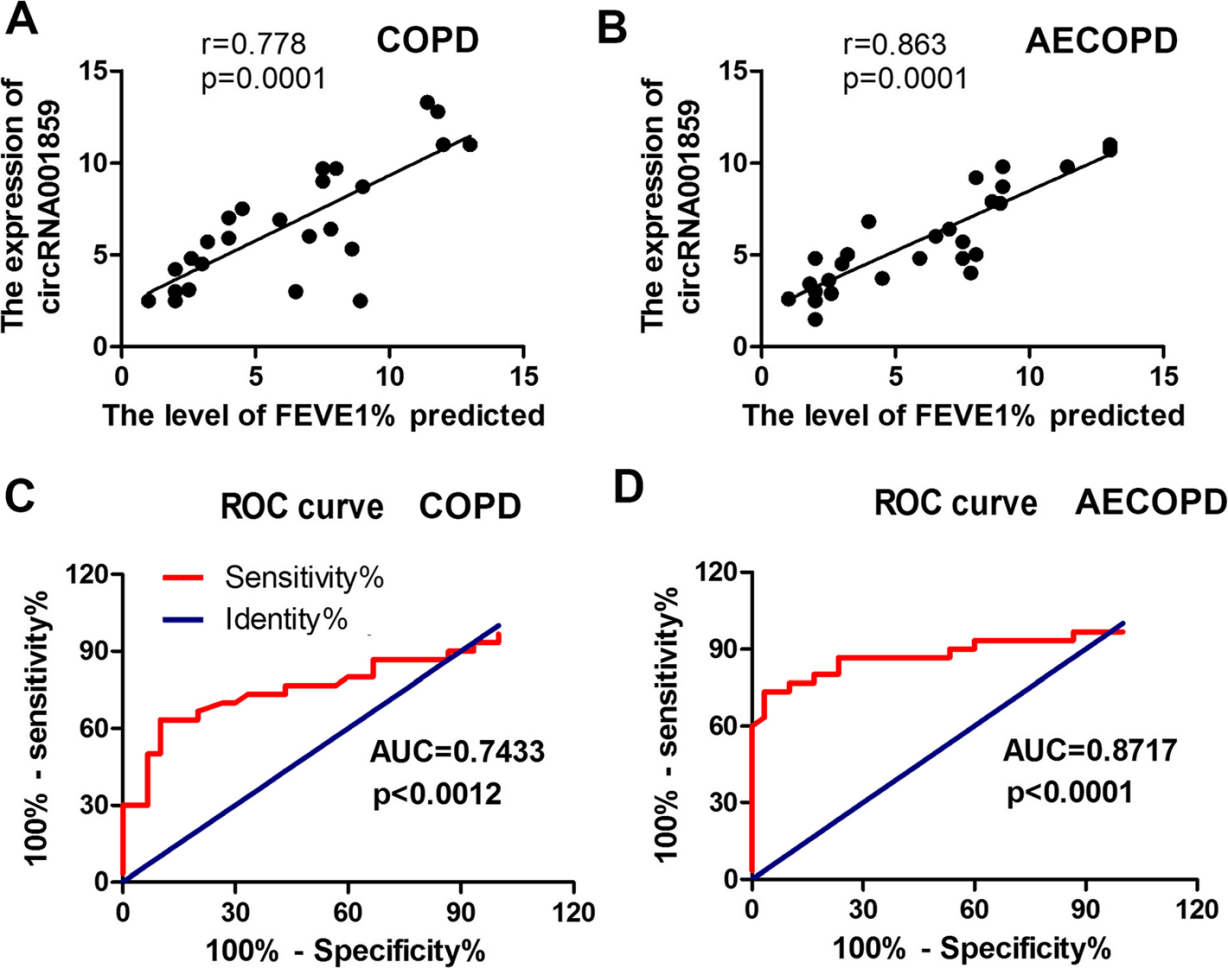
CircRNA0001859 expression level was correlated with the transformation from COPD to lung cancer. Spearman correlation analysis was carried out between FEV1/FVC% and circRNA0001859 expression in blood of (**a**) CDOP and (**b**) AECOPD patients. **c** ROC curve analysis of circRNA0001859 expressions for predicting stable COPD risk from HCs; **d** ROC curve analysis of circRNA0001859 expressions for predicting AECOPD risk from HCs. Three independent biological repeats were carried out for each analysis

We analyzed the correlation between circRNA0001859 expression and the factor of lung function FEV1% in COPD and AECOPD patients. The results indicated a positive correlation between circRNA0001859 expression and FEV1% both in the COPD and AECOPD patients (Fig. 6a, b). On receiver operating characteristics (ROC) curve analysis, serum circRNA0001859 reflected obvious distinction between the control and COPD groups, with areas under the curve of 0.7433, 08717; sensitivity of 41.6, and 17.8%; and specificity of 95.8 and 95.4%, respectively (Fig. 6c, d).

## Discussion

A Number of biomacromolecules have been dig out that has the possibility to be the diagnostic molecular such as miR-125b, miR-146a/b, mir-190a-5p and miR-210 [20–23]. However, none of them has been applied to clinical use. The unique of stable structure, good conservation, tissue specificity as well as the vital biological functions make circRNAs idea molecular as the prognosis biomarkers [24]. CircRNAs have been seldom studied previously. It was only reported that hsa _circ_0016070 was identified to be associated with the risk of PAH in COPD patients [25]. In addition, a unique set of circRNAs and mRNAs expression patterns were found in primary HSAECs treated with CSE and these dysregulated circRNAs might participate in airway cells response to cigarette smoke-induced stress by post-transcriptional regulation of ceRNA networks [26].

It was demonstrated that CS exposure alters systemic mechanistic pathways, such as oxidative stress, metabolic alterations, autonomic nervous system dysfunctions and hormonal changes [27]. Nicotine, the addictive constituent of tobacco and its derived carcinogenic nitrosamines triggers activation of nicotinic acetylcholine receptor (nAChR) nAChR alpha7 (α7) plays a crucial role in modulating the response to chronic CS that may alter susceptibility to associated lung diseases including fibrosis [28]. The pathophysiology of COPD includes variety of cell types, macrophages, neutrophils and T-lymphocytes. There is a correlation between COPD severity and macrophage numbers. Cigarette smokers and other irritants can induce macrophages to release inflammatory mediators, TNF-α (tumor necrosis factor-α), MCP1 (monocyte chemotactic protein-1), ROS (Reactive oxygen species) and neutrophils chemotactic factors, such as LTB_4_ (leukotriene B_4_) and IL (nterleukin)-8 [29].

In the present work, we succesfully established the COPD and AECOPD model. With microarray, FISH and qRT-PCR analyses, we found the dysregulated expression of circ0001859 in COPD. Considering the potential effect of circRNA0001859 in COPD progression, we speculated whether circRNA0001859 could be a prognostic molecule. The markers derived from lung tissue are not routinely available for clinical disease monitoring, whereas blood is readily accessible. Thus, we evaluated the level of circRNA0001859 in the blood of mice and human. Consistently, we found that circRNA0001859 is decreased in the blood of COPD mice and patients compare to normal and healthy controls. In addition, the level of circRNA0001859 was lower in CS + NNK treated mice in comparison with that in CS treatment alone group. These findings indicated the critical role of circRNA0001859 in diagnose of the COPD.

Previously, using functional analysis, a total of 2132 circRNAs and 2734 mRNAs are dysregulated in COPD patients as compared to the normal controls; the ten most upregulated circRNAs in COPD patients are has_circ_0007294, has_circ_0004903, has_circ_0003003, has_circ_000157, has_circ_0001853, has_circ_0043926, has_circ_0002136, has_circ_0026466, has_circ_0051433, has_circ_0000364 and the ten most downregulated circRNAs are has_circ_000074, has_circ_0025453, has_circ_0056158, has_circ_0075043, has_circ_0077995, has_circ_0051785, has_circ_0031660, has_circ_0001744, has_circ_0043144, has_circ_0064900 [30]. Furthermore, has_circ_0008672 sponges miR-1265 and regulates the downstream signaling molecule mitogen activated protein kinase 1 (MAPK1) and GO analysis predicted that has_circ_0008672 is at the core of three KEGG pathways (Nod-like receptor signaling pathway, natural killer cell mediated cytotoxicity and Th17 cell differentiation); dysregulated circRNAs are involved in biological and cellular processes, such as tolerance induction, cell death and apoptosis, MAPK kinase activation and signal transduction that can be associated with the pathogenesis of COPD. has_circ_0008672/miR1265/MAPK1 axis regulates pathogenesis of COPD. RNA sequencing and bioinformatics analyses of the primary human small-airway epithelial cells (HSAECs) model of cigarette smoke extract induced-COPD revealed that 65 circRNAs are significantly upregulated and 100 circRNAs are dramatically downregulated in smoke group. Has_circ_0061052, circ_0011916 and circ_0002169 are upregulated while has_circ_0006892, has_circ_0006794 and has_circ_0008725 are downregulated [16]. Our findings extend our understanding of the role of circRNAs in COPD progression.

Here, the normal bronchial epithelial cells and exposed them to cigarette smoke and other chemicals. These cells may behave differently from bronchial epithelial cells obtained from COPD patients. It is the limitation of this study.

## Conclusion

In conclusion, we have investigated the potential role of a novel circRNA, circ0001859 in regulating COPD and AECOPD. Three treatment groups, CS or NK alone and CS + NNK were given to human and mice lung cells. Interestingly, circ0001859 was found to be downregulated under all the three treatment groups and under CS + NNK, circ0001859 was more downregulated then the CS or NNK alone. Our investigation has indicated that circ0001859 might act as a potential prognostic and diagnostic biomarkers for treating COPD and AECOPD.

## Data Availability

Data are available from the corresponding author upon reasonable request.

## Abbreviations

COPD: Chronic obstructive pulmonary disease
AECOPD: Acute Exacerbation of COPD
CS: Cigarette smoke
COVID-19: Coronavirus disease 2019
CircRNAs: Circular RNAs
PASMCs: Pulmonary artery smooth muscle cells
HSAECs: Human small-airway epithelial cells
CSE: Cigarette smoke extract
FBS: Fetal bovine serum
TNF-a: Tumor necrosis factor alpha
BALF: Bronchoalveolar lavage fluid
FISH: Fluorescence In situ Hybridization
ROC: Operating characteristi ccurve
SOD: Superoxide dismutase
